# Jejunal Tuberculosis Presenting as an Acute Intestinal Obstruction in a 74-Year-Old Patient: An Uncommon Encounter

**DOI:** 10.7759/cureus.77603

**Published:** 2025-01-17

**Authors:** Thanveer Suresh, Sudha Madhavan, Mohini Singh, S Ramakrishnan

**Affiliations:** 1 Internal Medicine, Sri Ramachandra Institute of Higher Education and Research, Chennai, IND

**Keywords:** acute abdomen, anti-tuberculosis therapy, granuloma, jejunal tuberculosis, small bowel obstruction

## Abstract

Abdominal tuberculosis involving the jejunum, although rare, can manifest with nonspecific symptoms, posing challenges in diagnosis and management, especially in elderly individuals.

The clinical course of a 74-year-old man who complained of acute onset vomiting, constipation, and abdominal discomfort is described in this case report. He was eventually found to have a small intestinal (jejunal) blockage as a result of abdominal tuberculosis (TB). The patient's significant unintentional weight loss over six months further complicated the clinical presentation. Radiological investigations, including abdominal X-rays, ultrasonography, and CT scans played a crucial role in identifying features suggestive of small bowel obstruction and guiding further management. Additionally, histopathological examination of the resected small bowel confirmed the diagnosis of necrotizing granulomatous inflammation, likely of tuberculosis origin. Management involved a combination of antituberculous therapy and surgical intervention. This example emphasizes how crucial it is to rule out abdominal TB when making a differential diagnosis for older patients who exhibit vague gastrointestinal symptoms, particularly in areas where the disease is prevalent or in those who have recognized risk factors. Timely diagnosis and multidisciplinary management involving clinical, radiological, and surgical expertise are essential for achieving favorable outcomes in such cases.

## Introduction

Abdominal tuberculosis (TB) is a relatively uncommon presentation of tuberculosis but is more prevalent in high-endemic areas like India, where 13% of all extrapulmonary TB cases are attributed to abdominal TB [1]. The most frequently involved sites are the peritoneum, intestines, and mesenteric lymph nodes [2]. Abdominal TB is acquired either by primary infection through ingestion, secondary infection via hematogenous/lymphatic dissemination, or reactivation of latent infection due to an immunosuppressed state [3]. Clinically, abdominal TB presents with symptoms like abdominal pain, weight loss, and altered bowel habits that overlap significantly with other gastrointestinal disorders, particularly Crohn’s disease [4]. The diagnosis of abdominal TB includes a combination of histopathological and microbiological investigations like AFB stain, cultures, radiological imaging, clinical judgment, and surgical intervention like diagnostic laparoscopy whenever necessary. One serious and perhaps fatal side effect of tuberculosis in the abdomen is acute small intestinal obstruction, which can be caused by a multitude of factors [5-7]. A strong index of suspicion is required for the diagnosis of small intestinal obstruction caused by tuberculosis, especially in endemic locations or in individuals who have recognized risk factors. Radiological investigations are essential in identifying the presence and extent of obstruction. Treatment of abdominal TB involves a combination of anti-tuberculous therapy (ATT) and surgical intervention when necessary.

## Case presentation

A 74-year-old male patient with a history of systemic hypertension, having undergone transurethral resection of the prostate, presented to the emergency clinic complaining of constipation and abdominal pain that had been present for the previous two days and one day of vomiting of three to five episodes which was bilious and contained food particles, while the abdomen pain was diffuse and severe in intensity. He claimed to have lost 12 kg of weight unintentionally over the previous six months. There were no associated symptoms such as fever, chest pain, palpitations, burning micturition, decreased urine output, diarrhea, night sweats, or cough with expectoration. He didn’t have any history of substance abuse and no other medical illness.

On examination, the patient was cachectic. Pallor was present. No icterus, cyanosis, clubbing, generalized lymphadenopathy, or pitting pedal edema was seen. BCG vaccination scar was noted on the left upper arm. Abdominal examination showed abdominal distension with diffuse abdominal tenderness and sluggish bowel sounds. There was no visible surgical scarring. Hernial orifices were free. Per rectal examination indicated normal tone with the presence of stools and no rectal mass. Other system examinations were unremarkable and lungs were normal.

Initial labs showed mild anemia with elevated ESR(103 mm/hr), normal CRP of 0.5mg/dl (normal less than 0.8 mg/dl), and hyponatremia, renal failure, albumin globulin reversal with other liver function tests were normal (Table 1).

**Table 1:** Initial laboratory investigations showing elevated ESR , mild anemia,hyponatremia and elevated serum creatinine.

Investigations	Patient’s value	Normal value
Hemoglobin	11.5 g/dL	12-17 g/dL
Total count	11,140/mm³	4000-11000 /cu.mm
Platelet	223,000/microlitre	150,000-450,000/microlitre
ESR	103	4-12
BUN	20 mg/dl	7-18mg/dl
Creatinine	1.9 mg/dl	0.6-1.3mg/dl
Sodium	120	134-144 mmol/L
Potassium	4.6	3.5-5 mmol/L
Chloride	99	96-108 mmol/L
Bicarbonate	21	21-29 mmol/L
Albumin	3.3	3.2-4.8 gm/dL
Globulin	5.8	2-3.5 gm/dl
Total bilirubin	0.62	0.1-1.2 mg/dl
AST	31	0-35 U/L
ALT	15	0-41 U/L

**Figure 1:**
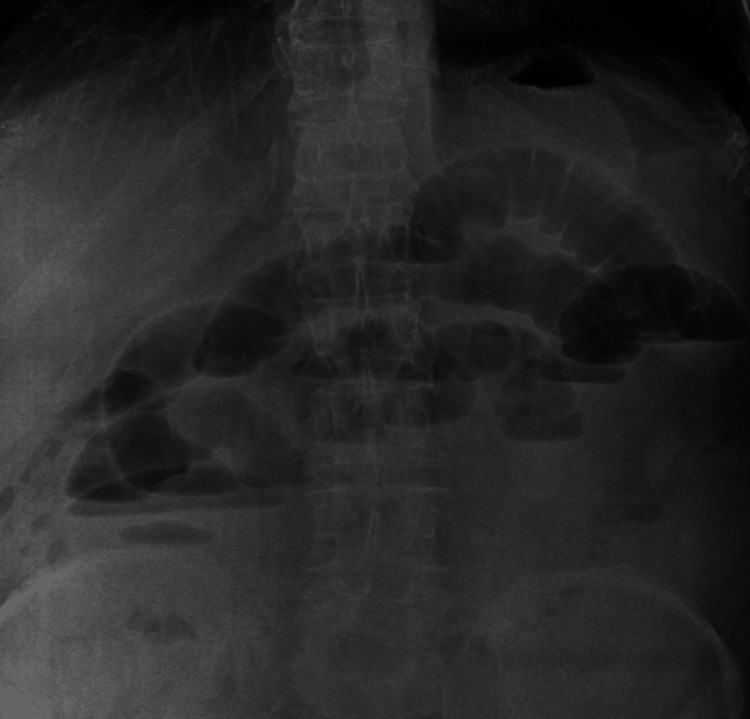
Abdominal X-ray erect image: showing dilated intestinal loops with air fluid levels.

Chest X-ray was normal. An Erect abdominal X-ray suggested dilated intestinal loops, with air-fluid levels (Figure 1).

**Figure 2:**
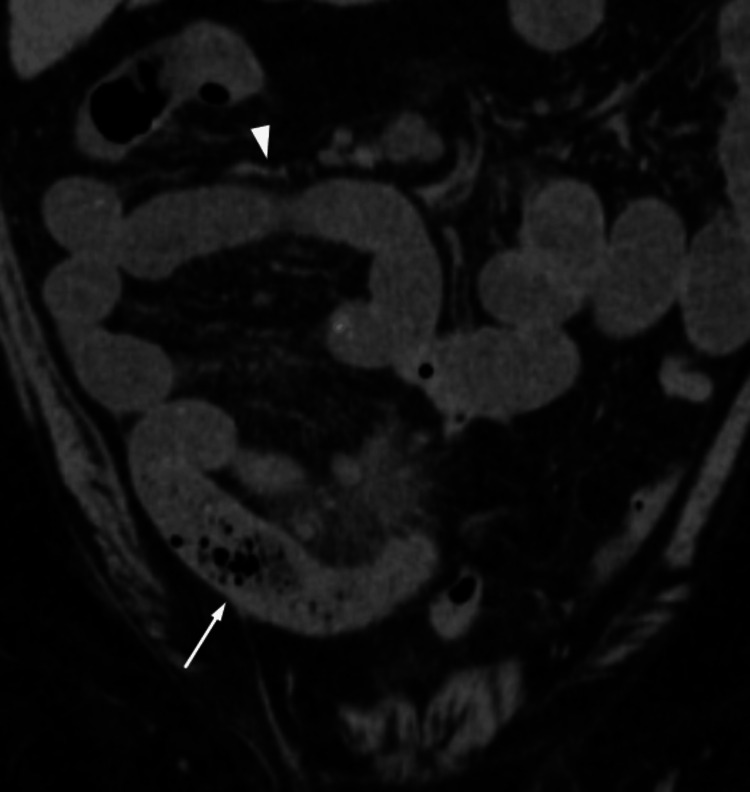
Abdominal CT scan image in coronal view showing dilated small bowel loops (white arrowhead) with the small bowel feces sign (white arrow). CT: computed tomography

At this stage, tuberculosis was suspected due to the patient's history of significant weight loss, constipation acute onset abdominal pain malignancy, and chronic infection. He was initially admitted to the ICU and was kept on nil per oral (NPO). A nasogastric tube was inserted for decompression and he was started on IV fluids and piperacillin-tazobactam. Carcinoembryonic antigen (CEA) was negative despite the suspicion of malignancy. A Mantoux test and a Quantiferon Gold assay were both positive. CT scans of the whole abdomen plain showed the whole jejunum and proximal ileum were dilated, and the mid-ileum displayed the small bowel feces sign, wall edema, mesenteric fat stranding, and surrounding fluid. The distal ileum and the colon seemed to have collapsed, suggesting a small intestinal obstruction with enlarged mesenteric lymph nodes (Figure 2).

**Figure 3:**
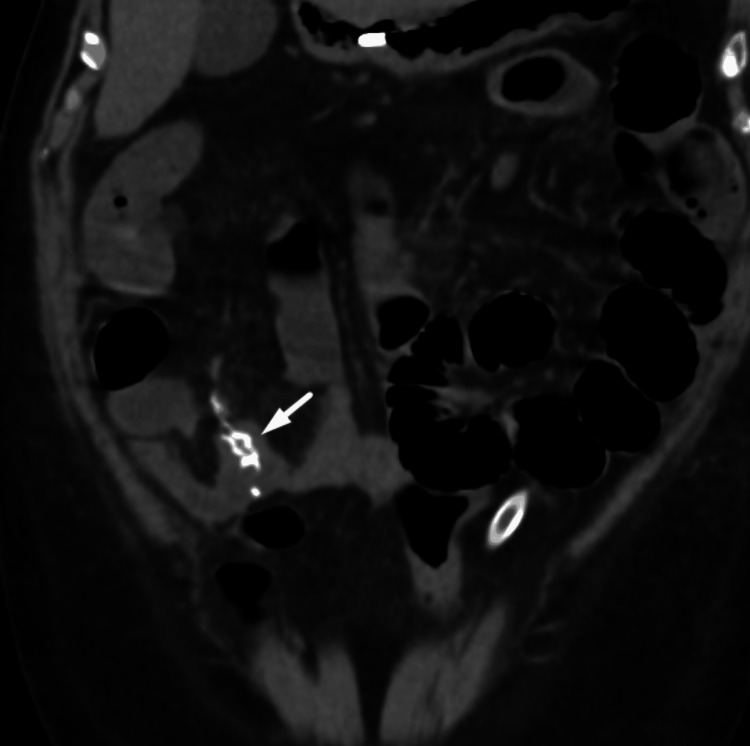
Abdominal CT scan image in coronal view showing anastamosis of the jejunum following resection of the jejunal growth (white arrow). CT: computed tomography

The patient had features of acute abdominal (small intestinal) obstruction and underwent an emergency laparotomy. During the procedure, a jejunal growth of 20 cm was noted 180 cm from the ileocecal junction, along with enlarged mesenteric lymph nodes. The surgical team proceeded with resection and anastomosis of the jejunal growth with a 5 cm margin on either side (Figure 3).

**Figure 4:**
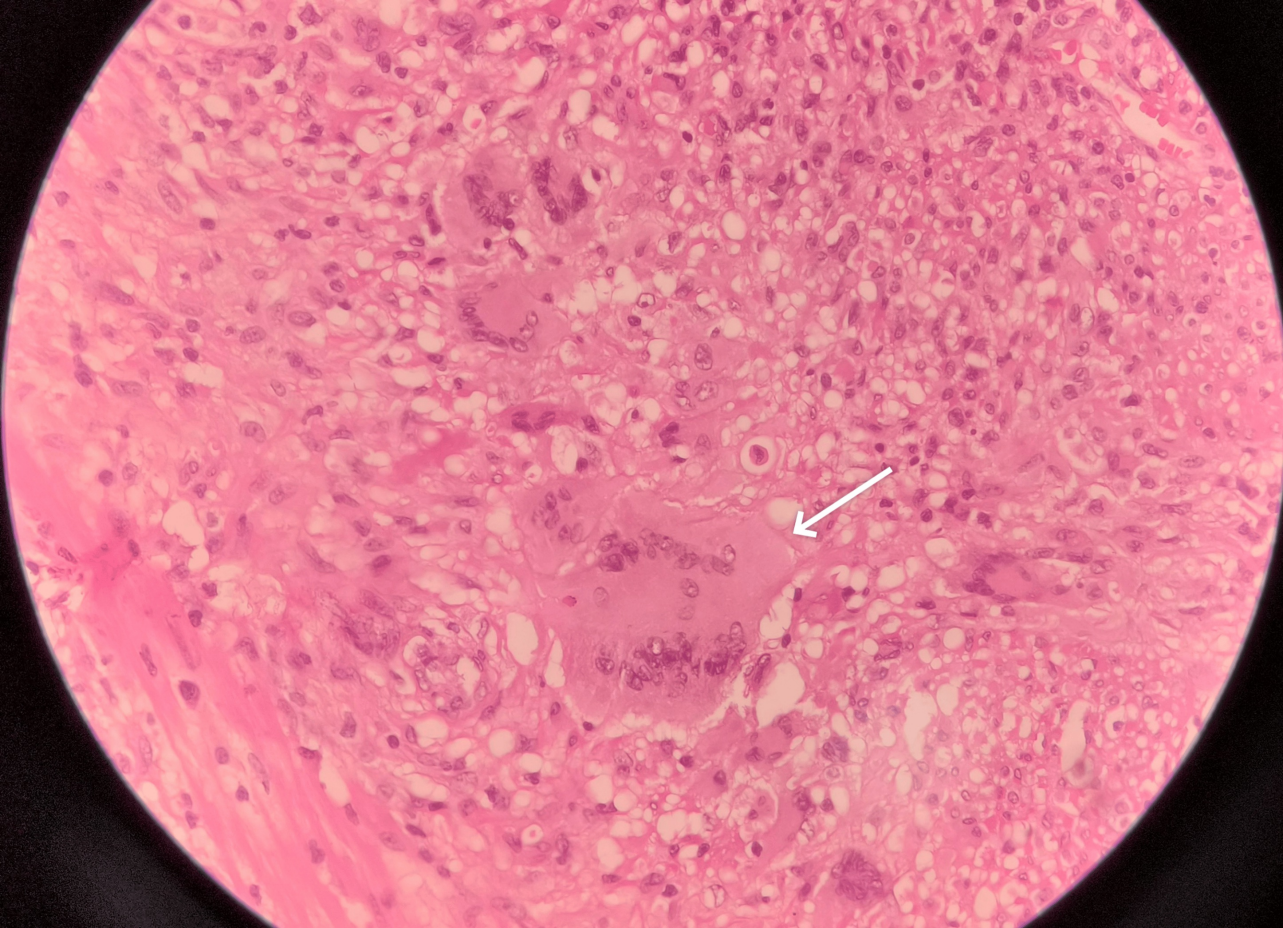
Histopathological examination of the resected small bowel showing langhan type multinucleated giant cell (white arrow).

The tissue was sent for histopathological examination, which showed bowel mucosa with extensive erosion and transmural inflammation extending to the serosa. Multiple epithelioid granulomas with Langhan-type multinucleated giant cells were present in the lamina propria, with focal areas of necrosis and numerous tingible body macrophages (Figure 4).

Perivascular lymphoplasmacytic infiltrates were also noted. Out of nine lymph nodes, seven showed granulomatous lymphadenitis. Special stains for fungi and acid-fast bacilli were negative.

Based on HPE findings of epitheloid granuloma and necrotizing lymphadenitis, the diagnosis was narrowed down to small bowel necrotizing granulomatous inflammation, likely of tuberculosis origin.

The patient was started on weight-based oral antituberculous therapy (ATT) based on histopathological examination findings. The intensive phase lasted for two months and consisted of daily doses of isoniazid 300 mg, rifampicin 600 mg, ethambutol 1200 mg, and pyrazinamide 1500 mg.

Postoperatively, the patient experienced persistent fever spikes and elevated creatinine levels. A CT KUB (kidneys, ureters, bladder) showed bilateral perinephric and periureteric fat stranding with normal-sized kidneys. Urine culture and sensitivity grew Klebsiella (10^5 CFU/mL). He was started on injections of piperacillin-tazobactam 4.5gm intravenously (IV) thrice daily for a total of 10 days, based on the culture sensitivity pattern, which only showed sensitivity to piperacillin-tazobactam. He showed clinical improvement and became afebrile. On POD 11, Ryle’s tube and drain from the surgical site were removed, and the patient was discharged.

During follow-up, the patient symptomatically improved. Following the intensive phase, he was continued on the maintenance phase of antituberculous therapy, consisting of daily doses of isoniazid 300mg, rifampicin 600 mg, and ethambutol 1200 mg, planned for four months duration.

## Discussion

The diagnosis and treatment of abdominal tuberculosis can be particularly complex since the illness can manifest with a wide variety of symptoms and consequences. The difficulties in identifying and treating abdominal tuberculosis are brought to light in this case report, particularly when the patient is elderly and has symptoms of small intestinal obstruction.

Pathogenesis of abdominal TB is diverse, encompassing primary infection through ingestion of contaminated food or water, reactivation of latent TB, and secondary spread from pulmonary TB via hematogenous or lymphatic dissemination [2]. In areas with high TB prevalence, abdominal TB often arises from the reactivation of latent infection due to immunosuppression or other predisposing factors. Immunocompromised states, including HIV infection, diabetes mellitus, malnutrition, and prolonged use of corticosteroids or immunosuppressive agents, significantly increase the risk of developing abdominal TB [3].

Clinically, abdominal TB presents with nonspecific symptoms that overlap significantly with other gastrointestinal disorders including Crohn’s disease, small bowel lymphoma, carcinoid syndrome, and neuroendocrine tumors. Patients may experience chronic or subacute abdominal pain, weight loss, anorexia, hyperthermia, night sweats, diarrhea, or constipation. The nonspecific nature of these symptoms often leads to delays in diagnosis, with patients frequently undergoing extensive evaluations for other potential causes of their symptoms [4]. Advanced disease may present with complications such as intestinal obstruction, perforation, abscess formation, or fistulae, necessitating prompt surgical intervention [5].

The gold standard for diagnosing TB continues to be histopathological inspection and microbiological investigations, such as acid-fast bacilli (AFB) staining and mycobacterial cultures. However, these tests often have low sensitivity in detecting abdominal TB, leading to reliance on clinical judgment and radiological findings [6]. Endoscopic procedures and tissue biopsies can provide critical diagnostic information, especially when radiological findings are inconclusive.

One of the most serious and potentially fatal consequences of abdominal TB is a small intestinal blockage that can be caused by strictures, adhesions, or external compression by swollen lymph nodes [7]. Since the ileocecal area has a lot of lymphoid tissue and a delayed transit time, which promotes the growth of mycobacteria, it is the most commonly afflicted location. However, jejunal obstruction secondary to tuberculosis occurred in our patient, which is rare and requires a high level of suspicion for the diagnosis, especially in endemic locations or in individuals who have recognized risk factors. Radiological tests, including CT scans, ultrasounds, and abdominal X-rays are crucial for determining the existence and degree of blockage as well as any related symptoms that may point to tuberculosis [8]. The patient may require diagnostic laparoscopy/explorative laparotomy and tissue samples should be sent for histopathology and AFB staining.

The treatment of abdominal TB involves a combination of ATT and surgical intervention when necessary. Standard ATT regimens are similar to those used for pulmonary TB, typically comprising a combination of isoniazid, rifampicin, ethambutol, and pyrazinamide [9]. Surgical intervention is indicated in cases with complications such as bowel obstruction, perforation, or abscess formation. Conservative surgery aimed at preserving bowel length is preferred, with procedures such as stricturoplasty or limited resection being favored over extensive bowel resection whenever possible [10].

Various studies collectively shed light on various aspects of abdominal TB, emphasizing its diagnostic challenges, clinical complexities, and management strategies.

Aregawi et al. (2022) showed that the terminal ileum and cecum are often impacted areas and that intestinal TB contributes to 2% of TB cases globally. The authors stressed that intestinal obstruction is a primary consequence that frequently presents with vague symptoms, requiring a high index of suspicion in order to make an early diagnosis and provide the necessary care [8]. Sasse et al. (2021) presented a case of intestinal TB with diagnostic challenges, highlighting the need for a multidisciplinary approach and early initiation of treatment based on clinical suspicion, even in the absence of microbiological confirmation [11]. Fu et al. (2020) underscored the diagnostic difficulties and significant morbidity and mortality associated with abdominal TB. Their case highlighted the importance of early diagnosis and timely treatment to manage the disease successfully and prevent complications [12].

Collectively, these studies contribute to a better understanding of the diverse presentations, diagnostic challenges, and management approaches for abdominal TB, highlighting the significance of early recognition, interdisciplinary collaboration, and swift initiation of treatment to improve patient outcomes and reduce morbidity and mortality associated with this condition. Intestinal obstruction secondary to ileocaecal tuberculosis is common while jejunal obstruction causing tuberculosis is very rare, a high index of suspicion is needed for early diagnosis like in our case to prevent morbidity and mortality.

## Conclusions

Abdominal TB, although rare, can lead to serious complications such as obstruction, emphasizing the need for prompt diagnosis and appropriate management. This case highlights the importance of challenges in diagnosing small intestinal obstruction (jejunum) secondary to TB particularly in the context of nonspecific symptoms and advanced age. A multidisciplinary approach, including clinical, radiological, and surgical expertise is crucial for achieving favorable outcomes in such cases.
